# Termination of Pregnancy Due to COVID-19 Induced Damage to the Placenta: A Case Report

**DOI:** 10.22088/cjim.13.0.295

**Published:** 2022

**Authors:** Shamsi Zare, Nasrin Sufizadeh, Payman Rezagholi

**Affiliations:** 1 Department of Obstetrics and Gynecology, Faculty of Medicine, Kurdistan University of Medical Sciences, Sanandaj, Iran; 2 Department of Operating Room, Faculty of Nursing and Midwifery, Kurdistan University of Medical Sciences, Sanandaj, Iran

**Keywords:** Termination of Pregnancy, COVID-19, Placenta damage

## Abstract

**Background:**

The effects of COVID-19 and its connection with pregnant women and infants have received growing attention of neonatal specialists and gynecologists. COVID-19 causes mild upper respiratory infections, leading to severe illness in patients with defective immune systems. In pregnant women with COVID-19 due to the adverse effects of this disease maintaining maternal health and preventing fetal death is essential and vital. The aim of this study is to report an unusual observation of Termination of pregnancy due to COVID-19 induced damage to the placenta.

**Case presentation:**

A 33-year-old female patient with a gestational age of 33 weeks. The main symptoms and main concerns of the patient were shortness of breath and cough. Following positive PCR test results and CT, the COVID-19 diagnosis was confirmed. Due to the positive OCT and fetal heart failure, it was decided to terminate pregnancy and thus the patient underwent emergency Cesarean section and the infant was born weighing 2700 g and Apgar 10.

**Conclusion:**

Common manifestations of COVID-19 in pregnant women include fever, cough, and muscle pain. The most common laboratory results are decreased blood lymphocytes and increased blood CRP. Pregnancy and childbirth complications in pregnant women with COVID-19 included elevated preterm delivery, increased Cesarean section rate, and infant mortality. As a result, pregnant women with COVID-19 should immediately have an ultrasound to diagnose placental thrombosis.

The impact of acute respiratory syndrome (COVID-19) and its association with pregnant women and infants has attracted considerable attention of neonatal specialists and gynecologists. Coronaviruses often provoke mild upper respiratory infections, which could be severe in immunocompromised individuals (1). Previous coronaviruses led to the outbreak of severe respiratory infections, such as Acute Respiratory Syndrome (SARS) from 2002 to 2004 and Middle East Respiratory Syndrome (MERS) from 2012 to the present. The SARS epidemic in 2003 and 2004 affected about 100 pregnant women (2). Most cases of severe SARS during pregnancy are associated with severe maternal respiratory infection, maternal death, and miscarriage (3). MERS, a common zoonotic disease transmitted by close human-to-human contact, infected about 1,845 people in 2012. In 5 cases of MERS during pregnancy, poor pregnancy and perinatal outcomes were reported. Pregnant women with COVID-9 demonstrate symptoms more severe than MERS and SARS. Still, adverse perinatal outcomes including elevated risk of miscarriage, preeclampsia, preterm birth, and stillbirth have also been reported (4).

In the cases of pregnancy with COVID-19, there may be abnormalities of placenta as opposed to the healthy pregnant women. Examples of these adverse events include intrauterine growth restriction, preeclampsia, preterm delivery, and stillbirth (5). According to the findings of Jeffrey Goldstein et al., increased monitoring during pregnancy may be necessary in patients with COVID-19 (6). The coronavirus epidemic, which emerged in 2003, affected about 100 pregnant women worldwide. Some studies have suggested severe maternal infection and the increased risk of miscarriage and maternal death among pregnant women with COVID-19. Studies also revealed that Middle East Respiratory Syndrome (MERS) is associated with adverse consequences for both mother and infant (7). A Chinese study on placenta reported massive perivillous fibrin deposition in placenta, multiple infarcts of chorionic villus, and chorangioma (8). In pregnant women, their tolerance to hypoxia decreases due to a weakened immune system and physiological changes in the respiratory system. Respiratory problems are expected to increase in pregnant women with COVID 19. But studies by researchers in China have shown that the clinical symptoms of pregnant women are not different from those of non-pregnant women. In pregnant women with COVID-19 due to the adverse effects of this disease such as intrauterine distress, premature rupture of membranes, abnormal amniotic fluid, abnormal umbilical cord and abnormal placenta, maintaining maternal health and preventing fetal death is essential and vital. The aim of this study is to report an unusual observation of termination of pregnancy associated with COVID-19. 

## Case Presentation

**Figure 1:**
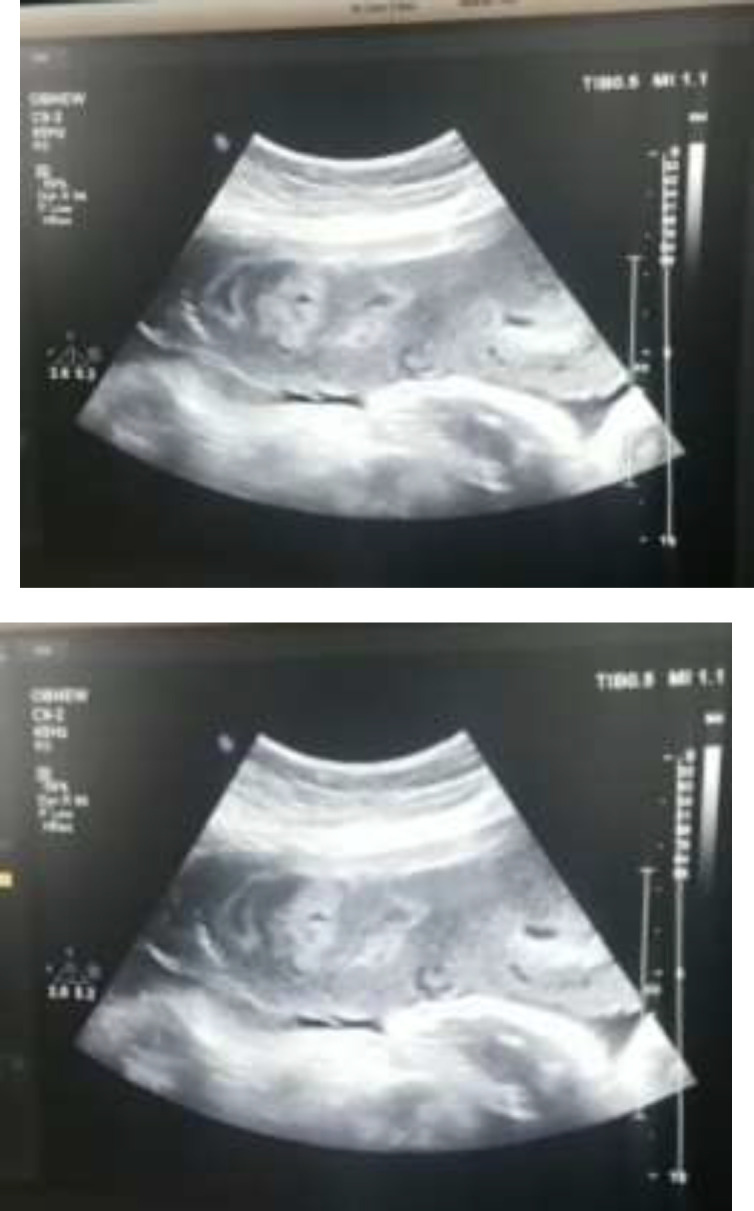
Ultrasound images of the placenta showing damage and fragmentation

**Figure 2:**
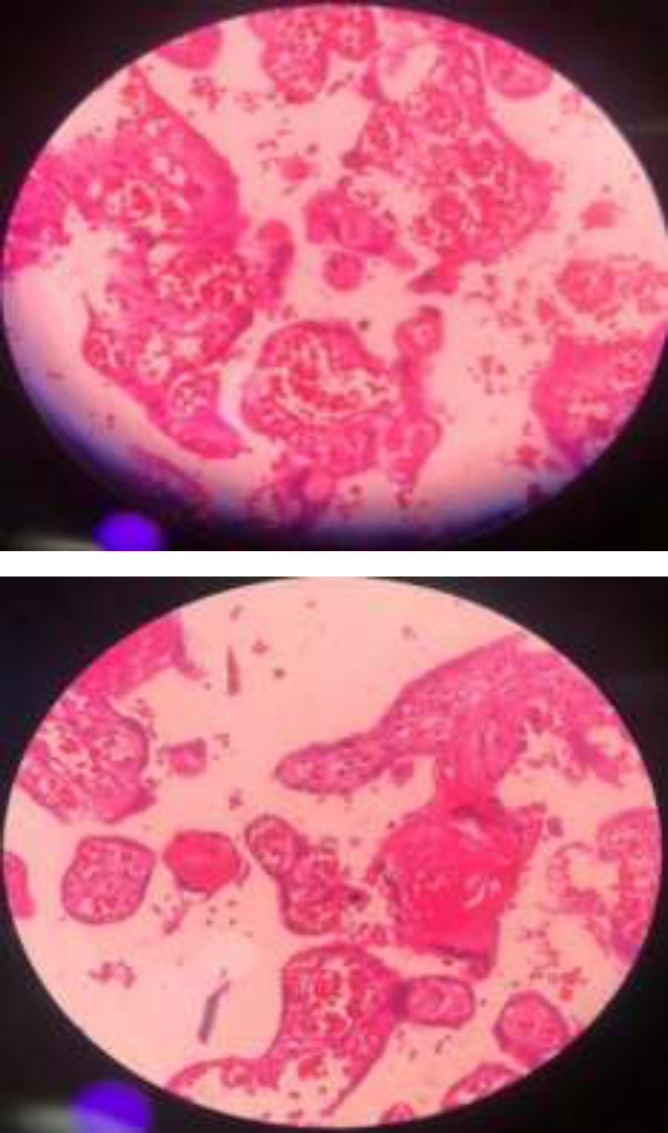
The pathology of the placenta that indicates vascular thrombosis

**Figure 3:**
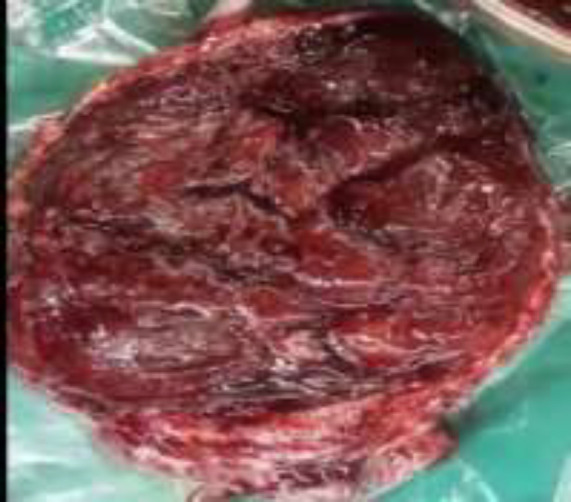
The image of the placenta has been extracted

This unusual case observation was reported after obtaining informed consent from the patient and receiving the code of ethics (IR.MUK.REC 1399.114) in Kurdistan University of Medical Sciences. The patient was a 33-year-old woman with a gestational age of 33 weeks, who had two previous abortions and has one child. The patient's BMI is 28.3 and has a history of Nephrectomy surgery and 2 pregnancies. The main symptoms and main concerns of the patient were shortness of breath and cough. On physical examination, a scar was seen on the right kidney and abdominal striae. The patient's vital signs are as follows: BP: 119/75 P: 82 SPO2:98 T: 36/2. Following the positive results of CRP test and CBC (WBC=8300 and PLT=201000) and CT scan, she was diagnosed with COVID-19 and discharged. The CT scan demonstrated multiple focal and GGO opacities in both lungs, indicating coronavirus pneumonia. Ultrasound showed normal AF, BPP = 8.8, positive PCR, and positive HRCT at the rate of 30%. After 19 days, she was re-admitted due to low FM and hypertension (Figure1). In view of positive OCT and fetal heart failure, doctors decided to terminate the pregnancy and the patient underwent emergency Cesarean section. Pfannenstiel incision was made on the skin accompanied by KER incision on the uterus and the infant was born weighing 2700 g and Apgar 10. The placenta was sent to the pathology for further examination (Figure3). The patient was discharged after 2 days with the prescription for enoxaparin 40 mg subcutaneous every 24hours until 6 weeks later and 6 weeks after discharge, he was evaluated for APS and was negative. In the macroscopic examination of the patient, the placental tissue was 14.5x14x2.9 cm in size and weighed 554 g, with an umbilical cord 32 cm long, 2 cm in diameter and 26 g in weight. In microscopic examination, moderate vascular thrombosis as well as damage to maternal and fetal vessels was observed (Figure2). 

## Discussion

The results of a study on nine women with COVID -19 in the third trimester of pregnancy revealed no fetal mortality, neonatal death or asphyxia, and in four cases, none of these outcomes could be attributed to COVID-19 infection (9), which is consistent with the findings of our report. In the study of Chen et al., all 9 mothers (9.9) underwent C-section and nasal cannula was placed for all subjects. Antiviral treatment and antibiotic treatment were prescribed for 6 of these mothers (67%) (9.9) (10) which is in line with the measures taken in our study. In the study by Zhu et al., a 6-day interval was reported between the onset of COVID -19 symptoms and the time of delivery. For seven pregnant women, C-section was performed and two had vaginal delivery. Prenatal problems including intrauterine distress (6 patients), premature rupture of membranes (5 to 7 h before the onset of actual labor (3 patients), abnormal amniotic fluid (2 patients), abnormal umbilical cord (2 patients) and abnormal placenta (1 patient) (11) were reported. 

In our study, the abnormalities of placenta were found, and the interval between the onset of COVID-19 symptoms and abortion was found to be 25 days. Yu et al. reported that of 11 deliveries, one was performed by vaginal delivery and 10 by C-section. The mean delivery time was 39 weeks and 2 days. Three patients underwent C-section at 34-36 weeks of gestation due to antiviral therapy. All patients received oxygen therapy through a nasal catheter (12). Chen et al. examined 9 infants born to mothers with COVID- 19. Of these neonates, 4 were preterm and 2 weighed less than 2500 g. In all of the newborns, 1 to 5 min after delivery, an Apgar score of above 8 was recorded. None of the infants experienced neonatal asphyxia and mortality (10) which is consistent with the results of the present study, though in this study infants were born with an Apgar score of 10. In the study of Zhu et al., out of 10 infants studied, six were preterm and three were born weighing less than 2500 g (11). 

In our study, the weight of the newborn was 2700 g. The findings of Lui et al. in relation to neonatal outcomes were as follows. In 10 mothers diagnosed with COVID-19, who gave birth to 11 infants (one had a twin), 90% of neonates were born by C-section and 4 were full-term. The administration of antiviral drugs in pregnant women was the reason for full-term pregnancy, but the experts decided to terminate the pregnancy (13). It is also consistent with the findings of the present study. The disadvantage (advantage or disadvantage?!) of this study is that with rapid diagnosis and treatment and emergency cesarean section despite placental thrombosis, the mother's life and fetal health were preserved. In this study, we had no limitations. 

In conclusion clinical signs, laboratory results, and radiographic criteria in pregnant women with COVID-19 resemble those of healthy adults. Common manifestations of COVID-19 in pregnant women included fever, cough, and muscle aches. The most prevalent laboratory results are decreased blood lymphocytes and increased blood CRP. Pregnancy and childbirth complications in pregnant women comprised increased preterm delivery and elevated rate of C-section. As a result, pregnant women with COVID-19 should immediately have an ultrasound to diagnose placental thrombosis. There is a paucity of studies in this field, which are mainly conducted in China. Hence, there is a need for further studies in this field in different countries worldwide.
